# Nutrient Regulation of Relative Dominance of Cylindrospermopsin-Producing and Non-cylindrospermopsin-Producing *Raphidiopsis raciborskii*

**DOI:** 10.3389/fmicb.2021.793544

**Published:** 2021-11-26

**Authors:** Lamei Lei, Minting Lei, Nan Cheng, Zhijiang Chen, Lijuan Xiao, Bo-ping Han, Qiuqi Lin

**Affiliations:** Department of Ecology, Jinan University, Guangzhou, China

**Keywords:** *Raphidiopsis raciborskii*, cylindrospermopsin, nutrients, co-culture experiment, dominance, real-time PCR

## Abstract

Raphidiopsis raciborskii (previously *Cylindrospermopsis raciborskii*) can produce cylindrospermopsin (CYN) which is of great concern due to its considerable toxicity to human and animals. Its CYN-producing (toxic) and non-CYN-producing (non-toxic) strains co-exist commonly in natural water bodies, while how their relative dominance is regulated has not been addressed. In this study, we combined field investigation with laboratory experiments to assessed the relationship between toxic and non-toxic *R. raciborskii* abundances under different nutrient levels. The *rpoC1*- and *cyrJ*-based qPCR was applied for quantifying total and toxic *R. raciborskii* abundances, respectively. The field survey showed that toxic *R. raciborskii* was detected in 97 of 115 reservoirs where its proportion ranged from 0.3% to 39.7% within the *R. raciborskii* population. Both total and toxic *R. raciborskii* abundances increased significantly with trophic level of these reservoirs, consistent with our monoculture and co-culture experiments showing in an increase in *R. raciborskii* growth with increasing nitrogen (N) or phosphorus (P) concentrations. In the monoculture experiments, growth rates of non-toxic and toxic strains from Australia or China were not significantly different under the same culture conditions. On the other hand, in the co-culture experiments, the toxic strains displayed a significantly faster growth than non-toxic strains under nutrient-replete conditions, resulting in an obvious shift toward the dominance by toxic strains from day 3 to the end of the experiments, regardless of the strain originating from Australia or China. The reverse was found under N- or P-limited conditions. Our results indicated that the toxic strains of *R. raciborskii* have a competitive advantage relative to the non-toxic strains in a more eutrophic world. In parallel to an increase in dominance, both toxic strains grown in the mixed population significantly increased CYN production under nutrient-replete conditions as compared to nutrient-limited conditions, suggesting that CYN may be of significance for ecological advantage of toxic *R. raciborskii*. These results highlight the importance of nutrient availability in regulating abundances and strain dominance of two genotypes of *R. raciborskii*. Our findings demonstrated that elevated nutrients would favor the growth of CYN-producing *R. raciborskii* and CYN production, leading to more blooms with higher toxicity at global scale.

## Introduction

Cyanobacterial blooms are a global environmental concern due to their potential to produce a variety of cyanotoxins, namely microcystins (MC), nodularins, cylindrospermopsin (CYN) and neurotoxins (Buratti et al., 2017). Cyanotoxins occurring in freshwater threaten human health and have a severe impact on water quality and ecosystem functioning (El-Shehawy et al., 2012; Buratti et al., 2017). In natural freshwater bodies, a cyanobacterial bloom always contains mixtures of morphologically indistinguishable toxic and non-toxic strains or different genotypes of the same cyanobacterial species (Ha et al., 2009; Burford et al., 2014). The proportion and the abundance of toxic strains could determine cyanotoxin concentrations, representing a prominent indicator for health toxicological risk evaluation (Briand et al., 2008; Ha et al., 2009; Buratti et al., 2017). Hence, understanding how the dynamics of toxic and non-toxic strains is regulated is crucial for predicting cyanobacterial bloom toxicity.

Global warming and eutrophication related to excessive anthropogenic nutrients (primarily nitrogen and phosphorus) have been identified as two primary drivers influencing cyanobacterial proliferation (Paerl and Huisman, 2008; Paerl, 2018). Similarly, temperature and nutrients can also affect the dynamics of toxic and non-toxic cyanobacterial strains. An increase in temperature, nitrogen (N) and phosphorus (P) concentrations was found to individually or concurrently make toxic strains outcompete non-toxic ones for the same cyanobacteria species (Yoshida et al., 2007; Davis et al., 2009, 2010; Ha et al., 2009; Dziallas and Grossart, 2011; Conradie and Barnard, 2012; Lei et al., 2015; Wang et al., 2018). These studies predict that climate change and further eutrophication will alter the community composition and promote blooms dominated by toxic cyanobacterial strains. However, several other studies found that non-toxic strains would become prevailing under the environmental conditions that favored cyanobacterial growth (Briand et al., 2008; LeBlanc Renaud et al., 2011). Despite these previous studies, how the environmental factors affect the dynamics of toxic and non-toxic cyanobacterial strains remain to be answered, due to their inconsistencies.

*Raphidiopsis raciborskii* is one of the most successful bloom-forming cyanobacteria in freshwater (Antunes et al., 2015; Burford et al., 2016). This organism is able to produce diverse cyanotoxins such as CYN, saxitoxins and other unidentified toxins, while CYN is the most commonly reported one for this species (Chiswell et al., 1999; Rzymski and Poniedziałek, 2014). Exposure to CYN has been shown to increase the production of reactive oxygen species (ROS) and may result in serious cytotoxicity, genotoxicity and reproductive toxicity, representing a serious hazard for human and animal health (Buratti et al., 2017; Scarlett et al., 2020). Traditionally considered a tropical species, its recent expansion toward temperate regions has been attributed to increasing water temperatures related to global warming (Sinha et al., 2012; Sukenik et al., 2012). The physiological plasticity and/or diverse nutritional strategies are as well critical for its growth and expansion (Antunes et al., 2015; Burford et al., 2016). Therefore, *R. raciborskii* has been shown to prevail over a wide range of nitrogen and phosphorus concentrations (Moisander et al., 2012; Chislock et al., 2014; Burford et al., 2016; Recknagel et al., 2019). However, these studies didn’t distinguish between toxic and non-toxic strains and only concerned *R. raciborskii* population.

Comparative genomic analysis has shown that the obvious genetic difference between CYN-producing and non-CYN-producing *R. raciborskii* was the presence or absence of the CYN biosynthesis gene cluster (Sinha et al., 2014). Additionally, two genotypes also exhibited differential physiological responses to environmental conditions. Due to the differences in structure and functioning of the photosynthetic apparatus between a toxic and non-toxic strain, Pierangelini et al. (2014) demonstrated that different light conditions may regulate succession patterns of two genotypes and affect bloom toxicity. A recent study demonstrated the physiological superiority of toxic strain over non-toxic strain under N-deficient and Fe-limited conditions (Fu et al., 2019). Several field studies have observed a seasonal succession within two genotypes of *R. raciborskii*. Orr et al. (2010) and Al-Tebrineh et al. (2012) found that though CYN-producing *R. raciborskii* strains dominated in the initial stage, the non-toxic strains became predominant toward the end of the bloom in several Australian freshwater bodies. The authors proposed that the proportion of toxic and non-toxic strains could explain the varying bloom toxicity. Nutrients could as well play an important role in shifting the strain dominance of *R. raciborskii.* Higher NH_4_-N concentrations was found to favor the growth of *R. raciborskii* leading to higher abundances (*pks* genes) of toxic strains in the Macau storage reservoir (Zhang et al., 2014). Recently, two mesocosm experiments definitely revealed the relationship between the nutrients and the relative dominance of toxic and non-toxic strains of *R. raciborskii* (Burford et al., 2014). Burford et al. (2014) suggested that toxic strains responded more quickly than non-toxic strains to the addition of P alone or with NO_3_, resulting in a shift toward the dominance of the toxic ones. In addition, an increase in CYN quota was also observed in such treatments (Burford et al., 2014). Bar-Yosef et al. (2010) have demonstrated a possible ecological role of CYN in phosphorus acquisition, which could offer potential competitive advantage to the CYN-producer *Aphanizomenon ovalisporum*. These limited studies suggest that nutrients might drive the dynamics of CYN-producing and non- CYN-producing strains of *R. raciborskii*, consistent with previous findings in *Microcystis* (Davis et al., 2009, 2010; Ha et al., 2009; Wang et al., 2018). Nevertheless, compared with *Microcystis*, competitive dynamics between the two genotypes of *R. raciborskii* are still poorly investigated.

Our previous study showed that CYN-producing *R. raciborskii* was distributed in 34 of the 46 (sub)tropical reservoirs of southern China and its proportion within *R. raciborskii* population varied from 0.3 to 34.7% (Lei et al., 2019). The fact that the non-toxic strains dominated in this region may assumed that the non-toxic genotype had a competitive advantage across the range of environments within the reservoirs. However, this assumption was obviously inconsistent with the findings in Australian freshwater bodies, where toxic strains frequently dominated within *R. raciborskii* population in the reservoirs (Willis et al., 2016b). A seasonal succession of toxic/non-toxic strains and nutrient-related dominance of toxic *R. raciborskii* have also been found (Orr et al., 2010; Al-Tebrineh et al., 2012; Burford et al., 2014). Several recent studies revealed that a high ecophysiological variability observed within and between locally occurring *R. raciborskii* strains (Bolius et al., 2017; Xiao et al., 2017; Fu et al., 2019; Baxter et al., 2020). Thus, investigating whether Chinese and Australian strains differentially respond to environmental conditions may provide an insight into the key driver promoting the dominance of the toxic *R. raciborskii.*

Based on these backgrounds, here we hypothesized that nutrients may be primary factors controlling the abundance and proportion of toxic *R. raciborskii* in natural waters. In order to test this hypothesis, the data from 115 reservoirs of Guangdong province in southern China was initially collected to assess how the nutrient levels regulated the abundance and proportion of toxic *R. raciborskii.* Furthermore, co-culture experiments were conducted under different nutrient concentrations to estimate whether the relative dominance of toxic and non-toxic strains was nutrient-related. We used two pairs of toxic and non-toxic strains of *R. raciborskii* from China (Guangdong province) and Australia respectively to evaluate whether locally occurring strains responded differently to nutrient changes. The CYN production was also determined to find out if this toxin played a role in the competitive dominance of toxic *R. raciborskii* strains.

## Materials and Methods

### Field Sample Collection

Field samples were taken from the reservoirs of Guangdong Province located at the transition between tropical and subtropical zones in southern China (Supplementary Figure 1). Due to the lack of natural lakes, many reservoirs have been constructed for drinking water supplies, aquaculture, recreation and agricultural irrigation. We sampled 46 and 69 reservoirs from September to November 2016 and from July to August 2018, respectively. For each reservoir a single water sample was taken from the zone in front of the dam. A total of 115 samples were collected.

At each site, water temperature and pH were measured *in situ* using a YSI-6600 (YSI Inc., United States). Water transparency was measured with a Secchi disk (SD). Surface water was taken with a 5-l UWITEC water sampler and transported to the laboratory on ice in a brown glass bottle. We measured total nitrogen (TN) and total phosphorus (TP) concentrations according to Chinese national standards for water quality and the methods by American Public Health Association [APHA] (1989). Chlorophyll-a (chl-a) concentration was determined spectrophotometrically after filtration (Whatman GF/C) and acetone extraction (Lin et al., 2005). The trophic state assessment of reservoirs was defined using the Carlson trophic state index (TSI) calculated with four variables: chl-a, SD, TN and TP (Carlson, 1977; Kratzer and Brezonik, 1981).

Phytoplankton samples collected from 0.5 m below the water surface were lightly filtered onto GF/C filters (Whatman) using vacuum filter for DNA extraction. Meanwhile, subsamples of 1 mL were taken for total CYN analysis. They were all stored at −80°C until DNA extraction or CYN detection.

### *Raphidiopsis raciborskii* Strains and Culture Conditions

Two CYN-producing strains (*R. raciborskii* CS506 and QDH7) and two non-CYN-producing strains (*R. raciborskii* CS510 and N8) were used in the monoculture and co-culture experiments. *R. raciborskii* CS506 and CS510 were from the Australian National Algae Culture Collection (ANACC), and the former strain could produce CYN and deoxy-CYN (Willis et al., 2015). The whole genomes of *R. raciborskii* QDH7 and N8, isolated from (sub)tropical reservoirs of Guangdong province (southern China), have been sequenced. It revealed that the CYN biosynthesis gene cluster was only found in *R. raciborskii* QDH7 (data not shown), which mainly produce deoxy-CYN detected by LC-MS/MS analysis (Lu et al., 2020). These four strains were non-axenic and regular microscopic inspection was used for confirming low bacterial abundances. All strains were maintained in BG11 medium (Rippka et al., 1979) at 25°C with irradiance of 60 μmol m^–2^ s^–1^ in a 12/12 h light/dark cycle. The illumination was supplied with cool white fluorescent tubes and light intensities were measured using a QSL2101 Scalar PAR Irradiance Sensor (Biospherical Instruments Inc., United States). The pre-cultures of each strain were manually shaken twice daily during incubation until the exponential phase.

### Monoculture Experiments

To investigate the effects of different nitrogen (N) or phosphorus (P) concentrations on the growth of four strains under the appropriate irradiance and temperature condition, the batch culture experiments were conducted. Cells cultured in complete BG11 medium with N concentration of 247 mg L^–1^ and P concentration of 7.13 mg L^–1^ were used as control (C). Three nitrogen (N) concentrations (N1 = 24.7 mg L^–1^, N2 = 2.47 mg L^–1^, N3 = 0.494 mg L^–1^) under P-replete condition (7.13 mg L^–1^) or three phosphorus (P) concentrations (P1 = 713 μg L^–1^, P2 = 71.3 μg L^–1^, P3 = 14.26 μg L^–1^) under N-replete condition (247 mg L^–1^) were set. Each strain was cultured under a 16/8 h light/dark illumination of 35 μmol m^–2^ s^–1^ at 28°C.

Prior to the experiments, cells in the late-exponential phase were centrifuged, washed and then suspended in sterile N- and P-free medium for 3 days. Subsequently, aliquots of resuspended cells were transferred to three identical 50mL capped test tubes (25 mm × 150 mm) containing 30 mL of the medium. The initial chlorophyll-a (chl-a) concentrations for each strain were around 20 μg L^–1^. These tubes were gently shaken twice daily and chlorophyll-a concentrations were measured daily using a TD-700 laboratory Fluorometer (Turner Designs, California, United States). The specific growth rates (μ, d^–1^) of four strains during the exponential growth phase were calculated according to the following equation (Burford et al., 2014): (ln B_2_-ln B_1_)/(t_2_-t_1_), where B_1_ and B_2_ were chl-a concentrations at time t_1_ and t_2_ respectively.

### Co-culture Experiments

Based on the monoculture experiments, different nutrient treatments could be roughly classified as either nutrient-replete (control, N1 and P1), or nutrient-limited (N-limited (N2 and N3) or P-limited (P2 and P3)) for *R. raciborskii* growth (Supplementary Figure 2 and Supplementary Figure 3). We ran co-culture experiments to investigate dynamics between CYN-producing and non-CYN-producing *R. raciborskii* strains under nutrient-replete (control), N-limited (N3) and P- limited (P3) conditions. Two pairs of strains from Australia (CS506/CS510) and China (QDH7/N8) were used for this experiment. The same experimental conditions as in monoculture experiments were applied.

After 3 days of N and P starvation, cell concentrations of *R. raciborskii* strains were counted under an Olympus microscope with 400 × magnification using a Sedgwick-Rafter chamber. CYN-producing and non-CYN-producing cells were inoculated at a cell ratio of 1:1 in 500 mL Pyrex Erlenmeyer flasks containing 400 mL of the medium. Each treatment was done in triplicate, and the flasks were gently shaken twice daily. Cultures were sampled every 3-4 days from day 1 (inoculation) until the end of the experiments and divided into subsamples for real-time PCR and total CYN measurement.

### DNA Extraction and Real-Time PCR

Phytoplankton cells from 200 mL water from the field samples or *R. raciborskii* cells from 10 mL cultures in the competition experiments were filtered through Whatman GF/C filters. Total DNA was extracted using a DNeasy Plant Mini Kits (Qiagen) according to the manufacturer’s instructions. DNA concentration and purity (*A*_260_/*A*_280_) were assessed using a Nanodrop ND-1000 spectrophotometer.

Cell concentrations of CYN-producing *R. raciborskii* and its proportions in the *R. raciborskii* population were determined using real-time PCR through two genes, including the RNA polymerase C1 gene (*rpoC1*) and the cylindrospermopsin synthetase gene (*cyrJ*). These two genes were used to quantify total and CYN-producing *R. raciborskii* cell numbers respectively, both in water samples and algal cultures. The specific primers and the probes (listed in Table 1) have been described previously (Lei et al., 2019).

**TABLE 1:** Primers and probes used for real-time PCR.

**Target gene**	**Primer/probe**	**Sequence (5′-3′)**	**Size (bp)**	**Reference**
*rpoC1*	CYL4-F3 CYL4-R3 TaqMan probe	aacgggttcgtcatagaggta ggctacaggtgctgctaactt FAM taacagaatcacgagttcgccgcc BHQ1	186	Lei et al., 2019
*cyrJ*	cyrJ –F1 cyrJ –R1 TaqMan probe	tgattcgccaacccaaagaa gatcgttcagcaagtcgtgt FAM cggagtaatcccgcctgtcatagatgc BHQ1	165	

*FAM, 6-carboxyfluorescein; BHQ1, black hole quencher-1.*

Real time PCR reactions were performed in 50 μL volumes containing 5 μL10 × buffer, 250 μM dNTP mixtures, 0.5 μL (10 pmol) of each primer, 0.5 μL (10 pmol) of each Taqman probe, 0.3 μL of NEB Hot Start Taq DNA polymerase, 5 μL of DNA template; and sterile MilliQ water to a final volume of 50 μL, using an ABI Prism 7,500 thermal real-time PCR cycler. Each sample was prepared in duplicate. The PCR conditions were as follows: 95°C for 15 s and 45 cycles of 94°C for 15 s, and 55°C for 45 s. Negative controls without DNA were as well included in each real-time PCR run.

To quantify the numbers of copies of *rpoC1* and *cyrJ* in filed samples and cultures, the amplicon of aforementioned two genes of *R. raciborskii* CS506 was cloned into the pMD18-T vector (TaKaRa, China) to serve as external standards (Lei et al., 2019). The standard curves were generated as a linear regression for the relationship between the cycle threshold (Ct) values and the logarithmic value of plasmid copy numbers. The obtained Ct values from water samples or cultures were converted into *rpoC1* and *cyrJ* gene numbers according to the regression equations. The proportion of CYN-producing *R. raciborskii* cells to total *R. raciborskii* population was determined by dividing the copy numbers of *cyrJ* gene by the copy numbers of *rpoC1* gene. The non-CYN-producing *R. raciborskii* cell numbers were obtained from the differences between *rpoC1* and *cyrJ* gene copy numbers.

### Total Cylindrospermopsin Analysis

One milliliter of water samples or culture cells in the co-culture experiments stored at −80°C were taken out and lysed by the freeze-thaw cycle. Insoluble cell debris was removed by centrifugation (20 627 g × 10 min). CYN concentrations in the supernatant were tested by a direct competitive ELISA using Cylindrospermopsin Plate Kit (Beacon Analytical Systems Inc., United States) in accordance with the manufacturer’s procedure. The absorbance at 450 nm was measured using a BIO-RAD iMARK Microplate Reader. Total CYN concentrations were calculated from a standard curve of semi-log relationship between relative absorbance and toxin concentrations using standards provided with ELISA kits. The contents were expressed as microgram equivalents of CYN per liter.

In the co-culture experiments, the CYN quotas (Q_CYN_) were calculated corresponding to the ratio between total CYN concentration and abundance (*cyrJ* gene copy numbers) of the CYN-producing strain as determined by real-time PCR.

### Statistical Analysis

For the field investigation, One-way analysis of variance (ANOVA) was used to analyze the relationships between TSI and total or toxic *R. raciborskii* abundances and the proportion of toxic strains. Statistical differences in the growth (chl-a concentrations and gene copy numbers for the monoculture and co-culture experiments respectively) of *R. raciborskii* were evaluated by repeated measures One-way ANOVA and Tukey’s test. The effects of nutrients on CYN quotas were as well investigated by One-way ANOVA. For the monoculture experiments, comparison of the specific growth rates (μ) between CYN-producing and non-CYN-producing strains within CS506/CS510 or QDH7/N8 pairwise was tested by Two-way ANOVA. All analyses were performed with a SPSS 16.0 statistical package using a significance level of 0.05.

## Results

### Trophic States of (sub)Tropical Reservoirs, the Abundance and the Proportion of Toxic *R. raciborskii*, CYN Concentrations and Their Relationships

**FIGURE 1:**
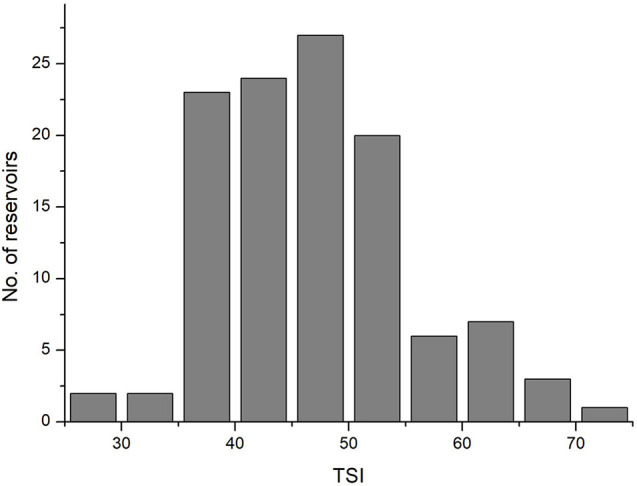
Histograms plot showing the summary statistics of TSI in 115 sampled reservoirs of southern China.

According to TSI values, only two reservoirs had an oligotrophic state (TSI < 30, Figure 1). Thus 115 reservoirs were classified as either oligo-mesotrophic (78 reservoirs, TSI < 50), or eutrophic (37 reservoirs, TSI > 50). The TSI values of oligo-mesotrophic and eutrophic reservoirs varied from 28.6 to 49.69 and 50.5 to 71.01, with an average of 42.05 and 56.54 respectively (Figure 1 and Table 2). Distinct difference in water transparency (SD) and chl-a concentrations was also observed between two types of reservoirs (*P* < 0.05, Table 2).

**TABLE 2:** Mean (± SD) of environmental parameters in oligo-mesotrophic (*n* = 78) and eutrophic (*n* = 37) (sub)tropical reservoirs in southern China.

**Environmental parameters**	**Reservoirs**	
	**Oligo-mesotrophic**	**Eutrophic**
TP (mg L^–1^)	0.029 (0.018)	0.2 (0.28)
TN (mg L^–1^)	1.09 (0.54)	2.24 (1.86)
Chl-a (μg L^–1^)	10.98 (8.87)	84.37 (69.32)
Temperature (°C)	29.11 (2.88)	27.14 (4.56)
Ph	7.28 (0.76)	7.67 (0.74)
SD (m)	1.45 (1.44)	0.62 (0.23)
TSI	42.05 (4.84)	56.54 (5.78)
CYN (ng mL^–1^)	0.42 (0.28)	0.52 (0.34)

*TN, total nitrogen; TP, total phosphorus; SD, Secchi disk; TSI, trophic state index; CYN, cylindrospermopsin.*

**FIGURE 2:**
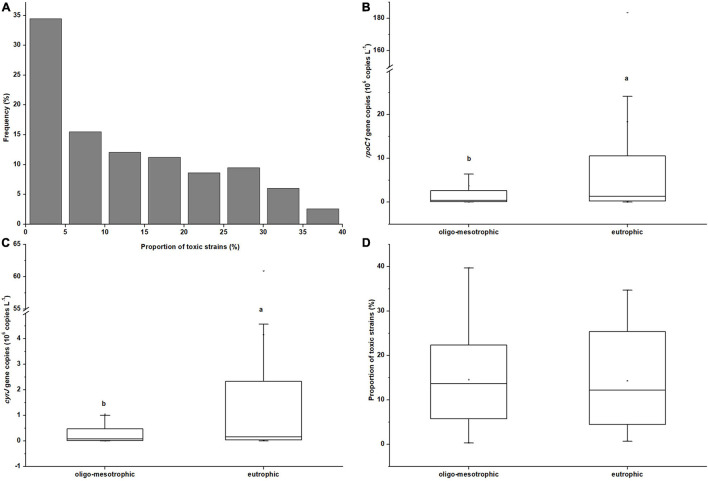
Histograms plot showing the summary statistics of proportion of toxic strains (*cyrJ*/*rpoC1*) within *R. raciborskii* population in 115 sampled reservoirs **(A)**; Box-whisker chart showing the summary statistics of total abundances of *R. raciborskii* **(B**, *rpoC1*gene copies), the abundances of its CYN-producing stains **(C**, *cyrJ* gene copies) and the proportion of toxic strains within *R. raciborskii* population **(D**, *cyrJ/rpoC1*) under oligo-mesotrophic (*n* = 78) and eutrophic (*n* = 37) (sub)tropical reservoirs in southern China. Letters denote significant differences between the two types of reservoirs.

The field survey showed that *R. raciborskii* (possessing *rpoC1* gene) was detected in all 115 (sub)tropical reservoirs with the real-time PCR. Its CYN-producing genotype (possessing *cyrJ* gene) also widely distributed in these reservoirs, with toxic strains appearing in 97 of 115 samples. The proportion of toxic strains (*cyrJ*/*rpoC1*) within *R. raciborskii* population ranged from 0.3% to 39.7%. Toxic strains had a relative abundance below 10% in nearly half of reservoirs and only 9 samples had a relatively higher proportion of toxic *R. raciborskii* (> 30%, Figure 2A), indicating a dominance of non-toxic strains in these (sub)tropical reservoirs.

The mean abundances of total and toxic *R. raciborskii* in eutrophic reservoirs were 1.83 × 10^7^ and 3.8 × 10^6^ cells L^–1^, which were significantly higher than those (3.73 × 10^6^ and 8.36 × 10^5^ cells L^–1^ respectively) in oligo-mesotrophic reservoirs (*P* < 0.05, Figures 2B,C). No significant difference was found between the proportions of toxic strains in these two types of reservoirs (*P* > 0.05, Figure 2D).

**FIGURE 3:**
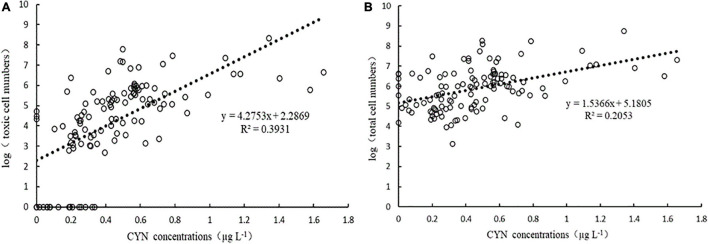
Correlation between CYN concentrations and the log_10_ toxic (*cyrJ* gene copies, **(A)** and total (*rpoC1* gene copies, **(B)** cell numbers of *R. raciborskii* determined by real-time PCR.

The samples from 10 reservoirs did not contain CYN and the CYN concentrations of 105 positive samples ranged from 0.1 to 1.657 μg L^–1^, among which 6 samples exceeded the guideline value of 1 μg L^–1^. CYN concentrations in these reservoirs showed a significant correlation with both the log_10_ abundance of total and toxic *R. raciborskii* (Figure 3). In a comparison of slopes (4.2753 and 1.5366) and coefficients of determination (*R*^2^ = 0.3931 and 0.2053) representing in Figures 3A,B, a higher correlation between CYN concentrations and toxic *R. raciborskii* abundances was found, suggesting a more suitable indicator for bloom toxicity.

### Growth of Cylindrospermopsin-Producing and Non- Cyn-Producing Strains Under Different N or P Concentrations in the Monoculture Experiments

Nutrient concentrations intensively affected the growth of both CYN-producing and non- CYN-producing *R. raciborskii* strains (Supplementary Figures 2, 3). The chl-a concentration curves of four *R. raciborskii* strains in N1 and P1 treatments were not significantly different from those in the control (*P* > 0.05). Significant differences between other treatments (N2, N3 or P2, P3) and the control were found (*P* < 0.05, Supplementary Figures 2, 3). This indicated that different nutrient concentration could be roughly classified as either nutrient-replete (control, N1 and P1), or nutrient-limited (N-limited (N2 and N3) or P-limited (P2 and P3)). The four strains exhibited a longer exponential phase and reached higher chl-a concentrations under nutrient-replete conditions than nutrient-limited treatments (Supplementary Figures 2, 3). Differential growth responses to nutrient changes were observed between Australian and Chinese strains. Both strain CS506 and CS510 exhibited a very short exponential period in the lowest N (N3) treatment (6 and 8 days for strain CS506 and CS510 respectively, Supplementary Figure 2A and Supplementary Figure 2C) while QDH7 and N8 did so in the lowest P (P3) treatment (Supplementary Figure 2 and Supplementary Figure 2).

**FIGURE 4:**
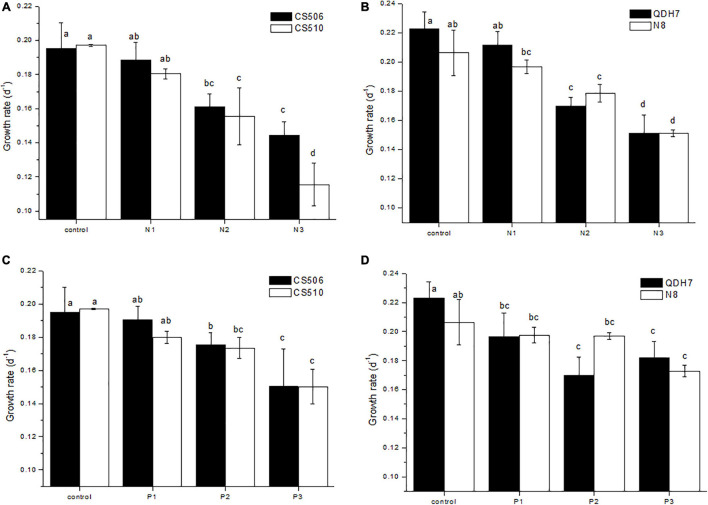
Specific growth rates (μ) for two Australian (CS506 and CS510) and two Chinese (QDH7 and N8) strains of *R. raciborskii* under different nitrogen **(A,B)** or phosphorus **(C,D)** concentrations in the monoculture experiments. Growth rates were calculated with chl-a over days 1-7, and statistical difference between treatments within strains, and between strains, was denoted by letters above columns.

According to μ calculated with chl-a concentrations over days 1-7, although some variation existed between toxic and non-toxic strain, no significant difference was observed between the two genotypes within CS506/CS510 or QDH7/N8 combination under all culture conditions (*P* > 0.05, Figure 4) except N3 treatment for the two Australian strains (*P* < 0.05, Figure 4A).

### Growth and Proportion of Cylindrospermopsin-Producing and Non-cylindrospermopsin-Producing Strains Under Nutrient- Replete and Nutrient-Limited Conditions in the Co-culture Experiments

**FIGURE 5:**
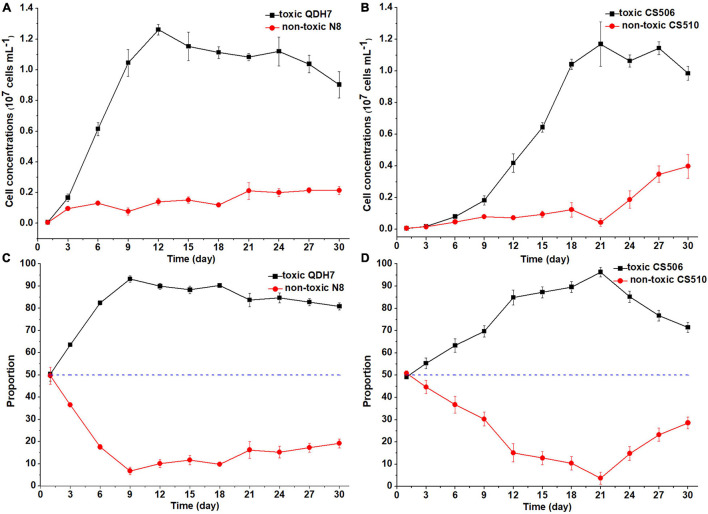
Changes in cell concentrations of toxic (*cyrJ* copy numbers) and non-toxic (the differences between *rpoC1* and *cyrJ* copy numbers) *R. raciborskii*, and proportion of toxic and non-toxic strains under BG11 control condition. Panels **(A,C)** showed data for QDH7/N8 co-culture experiments, whereas panels **(B,D)** showed data for CS506/CS510 co-culture experiments. These experiments were started with equal amounts of CYN-producing and non-CYN-producing cells (1:1, blue dotted line in panels **(C,D)**).

Under nutrient-replete conditions (BG11 control), both toxic strains QDH7 and CS506 in the mixed population rapidly increased, with maximum cell concentrations (1.26 × 10^7^ cells mL^–1^ and 1.17 × 10^7^ cells mL^–1^ respectively) appearing on day 12 and day 21 respectively, while non-toxic N8 and CS510 grew very slowly during the whole growth phase (Figures 5A,B). When the toxic strains entered stationary growth phase, both non-toxic strains still increased slowly. The non-toxic CS510 showed a faster growth from day 21 until the end of the experiments, with an increase in its proportion within the mixed population from 3.7 to 28.6% (Figure 5D). The *R. raciborskii* mixtures had an obvious shift toward dominance by the toxic strain (QDH7 or CS506) from day 3 and this advantage maintained until the end of the experiments (Figures 5C, D). The maximum proportion of toxic cells reached up to 93.2% and 96.3% for QDH7 and CS506 respectively, however, the non-toxic strains within *R. raciborskii* mixtures were not completely excluded by the toxic strains (Figures 5C, D).

**FIGURE 6:**
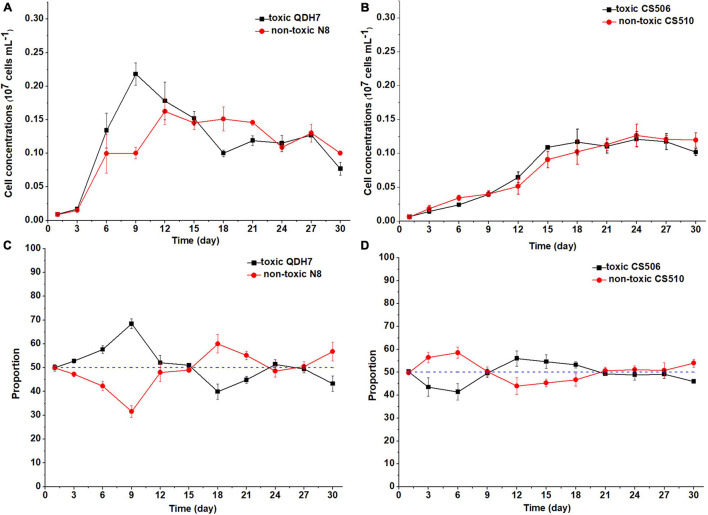
Changes in cell concentrations of toxic (*cyrJ* copy numbers) and non-toxic (the differences between *rpoC1* and *cyrJ* copy numbers) *R. raciborskii*, and proportion of toxic and non-toxic strains under N-limited (N3) condition. Panels **(A,C)** showed data for QDH7/N8 co-culture experiments, whereas panels **(B,D)** showed data for CS506/CS510 co-culture experiments. These experiments were started with equal amounts of CYN-producing and non-CYN-producing cells (1:1, blue dotted line in panels **(C,D)**).

**FIGURE 7:**
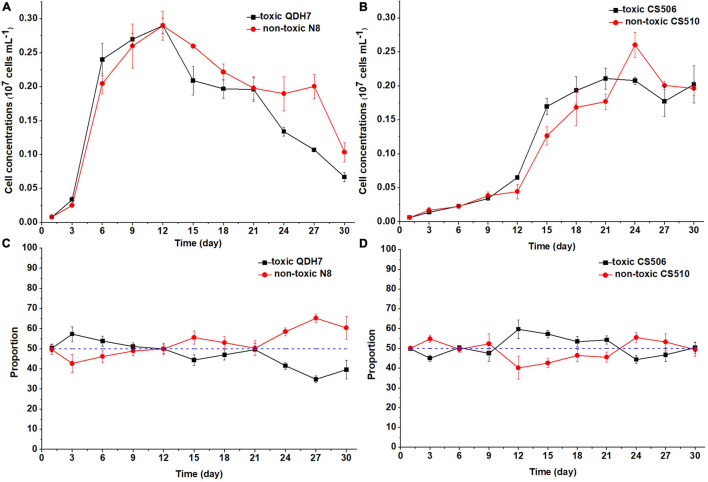
Changes in cell concentrations of toxic (*cyrJ* copy numbers) and non-toxic (the differences between *rpoC1* and *cyrJ* copy numbers) *R. raciborskii*, and proportion of toxic and non-toxic strains under P-limited (P3) condition. Panels **(A,C)** showed data for QDH7/N8 co-culture experiments, whereas panels **(B,D)** showed data for CS506/CS510 co-culture experiments. These experiments were started with equal amounts of CYN-producing and non-CYN-producing cells (1:1, blue dotted line in panels **(C,D)**).

Under nutrient-limited conditions, both toxic and non-toxic strains in the mixed population grew slowly, with maximum cell concentrations below 0.3 × 10^7^ cells mL^–1^ (Figures 6, 7). For Chinese strains, toxic QDH7 initially increased faster than non-toxic N8 especially under N-limited condition (Figures 6A, 7A), its proportion within *R. raciborskii* mixtures peaking (68%) on day 9 (Figure 6C). After 9 or 12 days, cell concentrations of both QDH7 and N8 simultaneously decreased and the mixed population gradually shifted toward dominance by the non-toxic N8 until the end of the co-culture experiments (Figures 6C, 7C). The maximum proportion of N8 strain were 60% and 65% under N-limitation and P-limitation, respectively (Figures 6C, 7C). For Australian strains, toxic CS506 and non-toxic CS510 showed a similar growth response to N or P limitation (Figures 6B, 7B). Although the relative dominance between the two strains displayed some fluctuation, their proportion in the mixed population remained approximately 50% throughout (Figures 6D, 7D).

### Cylindrospermopsin Production in the Co-culture Experiments

**FIGURE 8:**
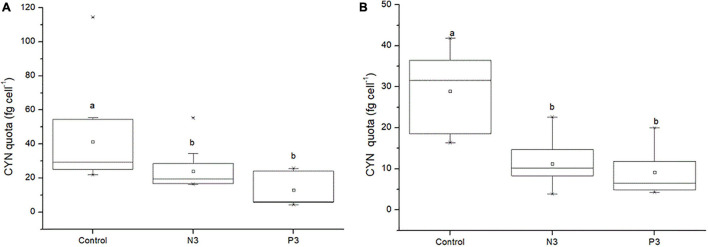
CYN quotas of *R. raciborskii* CS506 **(A)** and QDH7 **(B)** under BG11 control, N-limited (N3) and P-limited (P3) conditions in CS506/CS510 and QDH7/N8 pairwise co-culture experiments. Boxes represent 25th to 75th percentiles, horizontal line within box marks the median, and open square within box indicates the mean. Whiskers below and above the boxes indicated 10th and 90th percentiles, and asterisks represent 1th and 99th percentiles. Letters denote significant differences among treatments.

For the co-culture experiments, CYN quotas of both toxic strains (CS506 and QDH7) significantly decreased under N-limited (N3) and P-limited (P3) conditions as compared to the control (*P* < 0.05, Figure 8). There was no significant difference of CYN quotas observed between N3 and P3 conditions for both toxic strains (*P* > 0.05, Figure 8). The mean values of CYN quotas in the control were nearly 2-6 times as high as those under N3 and P3 conditions, respectively (Figure 8).

## Discussion

Our field investigation demonstrated that CYN-producing *R. raciborskii* widely occurred and commonly coexisted with non-CYN-producing strains in (sub)tropical reservoirs of southern China. The abundances of toxic strains positively correlated with trophic levels. Co-culture experiments with two pairs of CYN-producing and non- CYN-producing *R. raciborskii* strains showed that the toxic strains definitely dominated under nutrient-replete conditions but lost this advantage under nutrient-limited conditions, indicating that the toxic strains have a competitive advantage relative to the non-toxic strains in a more eutrophic world. These findings implied that nutrients may drive the dynamics of toxic and non-toxic *R. raciborskii.* This is the first study combining field investigation with laboratory experiments to explore how nutrients regulate strain composition within the *R. raciborskii* population.

To our knowledge, the present study represented the first attempt to differentiate the occurrence of toxic and non-toxic *R. raciborskii* in (sub)tropical reservoirs of southern China, in contrast to the numerous studies only regarding the *R. raciborskii* population (Bonilla et al., 2012; Lei et al., 2014; Antunes et al., 2015). By applying a previously established *rpoC1*- and *cyrJ*-based qPCR (Lei et al., 2019), we found that the proportion of *cyrJ* genotypes in the *R. raciborskii* population was no greater than 39.7% and even below 10% in nearly half of samples, suggesting that non-CYN-producing strains dominated in southern China. This finding was quite different from those observation in Australia and Europe. CYN-producing *R. raciborskii* frequently predominated in Australian water bodies (Fergusson and Saint, 2003; Orr et al., 2010; Willis et al., 2016b), while no European strains were found to produce CYN despite its common occurrence in this region (Fergusson and Saint, 2003; Rzymski et al., 2017). The contrasting results have already been indicated but not yet elucidated (Burford et al., 2016).

In the monoculture experiments, we found similar growth responses for toxic and non-toxic strains originating from the same country, indicating low variation within CS506/CS510 or QDH7/N8 pairwise, However, differential responses to nutrient changes were observed between locally occurring strains. Two Australian strains (CS506 and CS510) exhibited markedly poor growth in the lowest N (N3) treatment, while two Chinese strains (QDH7 and N8) did so in the lowest P (P3) treatment. Several recent studies showed the existence of multiple ecotypes with a high trait variability between and within locally occurring *R. raciborskii* strains (Willis et al., 2015, 2016b; Bolius et al., 2017; Xiao et al., 2017). Thus the four strains from two counties could be defines as two ecotypes with respect to nutrients.

The ability to fix atmospheric nitrogen has been proposed as a mechanism that allows *R. raciborskii* to bloom in nitrogen-limited freshwater bodies (Burford et al., 2016). For the strain CYP011K of *R. raciborskii*, nitrogen fixation has made it to grow with a higher growth rate under the combination of nitrate deprivation and phosphate repletion (Rigamonti et al., 2018). However, it seemed that N_2_ fixation can’t supply sufficient nitrogen for persistent growth of *R. raciborskii* under N-limited (N2 and N3) conditions as compare to N-replete conditions (control and N1) in our monoculture and co-culture experiments. Our result was in consistent with a previous study with the same two Australian strains which showed lower growth rates under N-fixing conditions than nutrient replete conditions (Willis et al., 2015). The process converting N_2_ to NH_4_^+^ is energy intensive (Dixon and Kahn, 2004). Thus nitrogen fixation may not be the principal mechanism for acquiring nitrogen and only result in a relatively low growth rate of *R. raciborskii* under N-deficient conditions (Burford et al., 2006; Willis et al., 2016a).

We found no significant difference in proportion of toxic *R. raciborskii* between oligo-mesotrophic and eutrophic reservoirs, and the non-toxic strains always dominated in these reservoirs regardless of trophic states. The results of the field investigation appeared to be inconsistent with those in the co-culture experiments showing that the higher nutrient concentrations led to an absolute advantage of toxic strains relative to the non-toxic strains. Given that the non-toxic strains lost dominance or maintained its proportion around 50% under N- or P-limited conditions in the co-culture experiments, we inferred that nutrient limitation may be proposed to explain this apparent inconsistency. Our field investigation demonstrated most of these (sub)tropical reservoirs were characterized as lower nutrients, especially P, similar to a previous study (Tang et al., 2019). This may confer a competitive advantage to non-toxic *R. raciborskii* rather than toxic ones due to the high cost of making CYN via a multienzyme complex (Neilan et al., 2013). The toxic strains may also have higher nutrient requirements than non-toxic ones due to the relatively high content of N in the molecular structures of the prominent cyanotoxins like MC and CYN (Vézie et al., 2002; Briand et al., 2012; Kiørboe and Andersen, 2019). Therefore, in nutrient-limited (sub)tropical reservoirs, non-toxic *R. raciborskii* always dominated in the population. We may expect that the *R. raciborskii* population in southern China will shift toward the dominance of the toxic strains as increased eutrophication.

As for monocultures, the growth rates over days 1-7 showed no significant difference between the two genotypes within CS506/CS510 or QDH7/N8 pairwise under most of the treatments. Correspondingly, if toxic and non-toxic strains were inoculated at a cell ratio of 1:1 subjected to the co-culture experiments, it might be expected that the proportions of two genotypes in the mixed population still maintain around 50%. However, the mixed population with two pairwise of *R. raciborskii* strains (CS506/CS510 and QDH7/N8) had an obvious shift toward dominance by the toxic strain (CS506 or QDH7) on day 3 under nutrient replete conditions. This finding indicated that the growth rates of the toxic strains were significantly higher than those of the non-toxic ones within *R. raciborskii* population. Some other studies also observed the disagreement between monoculture and co-culture experiments, and demonstrated that the existence of sympatric competitor was possibly responsible for this difference (Oberhaus et al., 2007; Briand et al., 2008). This phenomenon also could be explained by the allelopathy effect (Briand et al., 2008; Lei et al., 2015). Some previous studies showed that CYN may act as allelopathic substances to inhibit the growth of other competitors and assist in nutrient uptake, allowing the CYN producers to outcompete sympatric species in environments (Bar-Yosef et al., 2010; B-Béres et al., 2012; Rzymski et al., 2014; Chia et al., 2017; Rigamonti et al., 2018). Our findings of a coincidence in increased CYN synthesis and competitive advantage of toxic strains support that CYN production may be a functional strategy for competition with other phytoplankters. On the other hand, Pinheiro et al. (2013) found that CYN at environmentally occurring concentrations was unable to affect negatively the growth of microalgae. Other allelochemicals produced by *R. raciborskii* may also offer a competitive benefit and contribute to its stable dominance (Figueredo et al., 2007). Further experiments will be required to evaluate the allelopathic activity of our strains used in this study.

In our two co-culture experiments, CYN quotas in toxic *R. raciborskii* were higher under nutrient-replete conditions, consistent with that of Burford et al. (2014), who found P-replete treatments had higher CYN quotas. Conversely, some studies showed that unfavorable conditions such as N or P limitation may trigger higher CYN production (Saker and Neilan, 2001; Bácsi et al., 2006; Rigamonti et al., 2018; Yang et al., 2018; Nor et al., 2019). Our results were also different from several recent studies suggesting that CYN production was constitutive metabolic processes in toxic *R. raciborskii* and not affected by different light, CO_2_ and nutrient conditions (Pierangelini et al., 2015; Willis et al., 2015; Yang et al., 2017). However, all of these previous studies regarding the effects of environmental factors on CYN production were performed with *R*. *raciborskii* strains in monoculture. Our observation on CYN production of toxic *R. raciborskii* in the mixed population represented the conditions similar to natural waters where cyanobacterial blooms often consist of mixtures of toxic and non-toxic strains (Ha et al., 2009; Orr et al., 2010; Burford et al., 2014; Zhang et al., 2014; Pacheco et al., 2016). Differences in the behavior of toxic and non-toxic strains maintained in monocultures and the mixed cultures (mono versus mixed) have already been highlighted (Oberhaus et al., 2007; Briand et al., 2008). Therefore, a coincident increase in CYN production, toxic *R. raciborskii* abundance and its proportion under nutrient-replete versus nutrient-limited conditions demonstrated that nutrient availability can regulate the relative dominance of toxic and non-toxic strains of *R. raciborskii.*

## Conclusion

The first attempt to combine field investigation with laboratory experiments revealed that nutrient availability can regulate the relative dominance of CYN-producing and non-CYN-producing *R. raciborskii.* Nutrient limitation of (sub)tropical reservoirs of southern China may be responsible for the dominance of non-toxic *R. raciborskii*, while higher nutrients can shift *R. raciborskii* toward populations comprised of a larger proportion of toxic strains. A coincident increase in CYN production and relative dominance of toxic *R. raciborskii* suggested that CYN may be of significance for ecological advantage of toxic strains. We demonstrated that further eutrophication will produce more toxic *R. raciborskii* blooms and enhance bloom toxicity. In order to better understand whether higher nutrients will shift the *R. raciborskii* population toward the dominance of by toxic strains in the reservoirs of southern China, special mesocosm bioassays are required to investigate the effects of nutrient addition on the population dynamics.

## Data Availability Statement

The raw data supporting the conclusions of this article will be made available by the authors, without undue reservation.

## Author Contributions

LL wrote the manuscript. NC, ML, and ZC contributed to the accomplishment of the study. LX, B-PH, and QL performed the statistical analysis and wrote some sections. All authors contributed to manuscript revision, read, and approved the submitted version.

## Conflict of Interest

The authors declare that the research was conducted in the absence of any commercial or financial relationships that could be construed as a potential conflict of interest.

## Publisher’s Note

All claims expressed in this article are solely those of the authors and do not necessarily represent those of their affiliated organizations, or those of the publisher, the editors and the reviewers. Any product that may be evaluated in this article, or claim that may be made by its manufacturer, is not guaranteed or endorsed by the publisher.
