# Supplementary material for: Nutrient Regulation of Relative Dominance of Cylindrospermopsin-Producing and Non-cylindrospermopsin-Producing Raphidiopsis raciborskii
Source: Front Microbiol. 2021 Nov 26;12:793544. doi: 10.3389/fmicb.2021.793544 (PMC8664406; doi:10.3389/fmicb.2021.793544)
Supplement: Supplementary file 1 [file Table_1.DOCX]

**
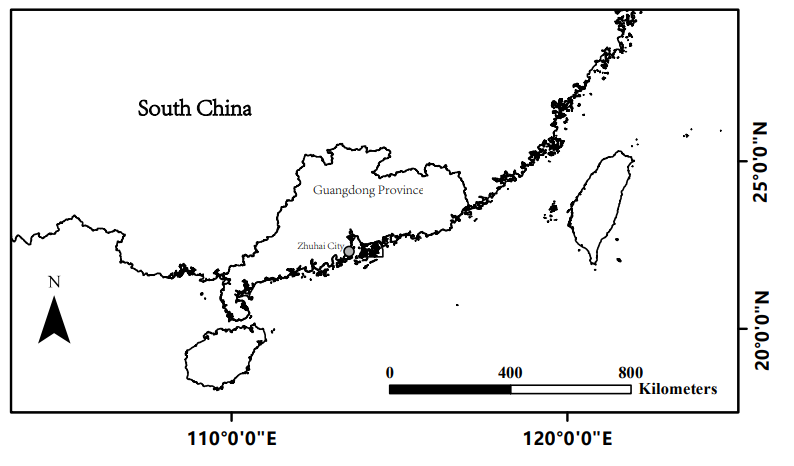
**

**FIGURE S1** Map of south China and location of Guangdong Province


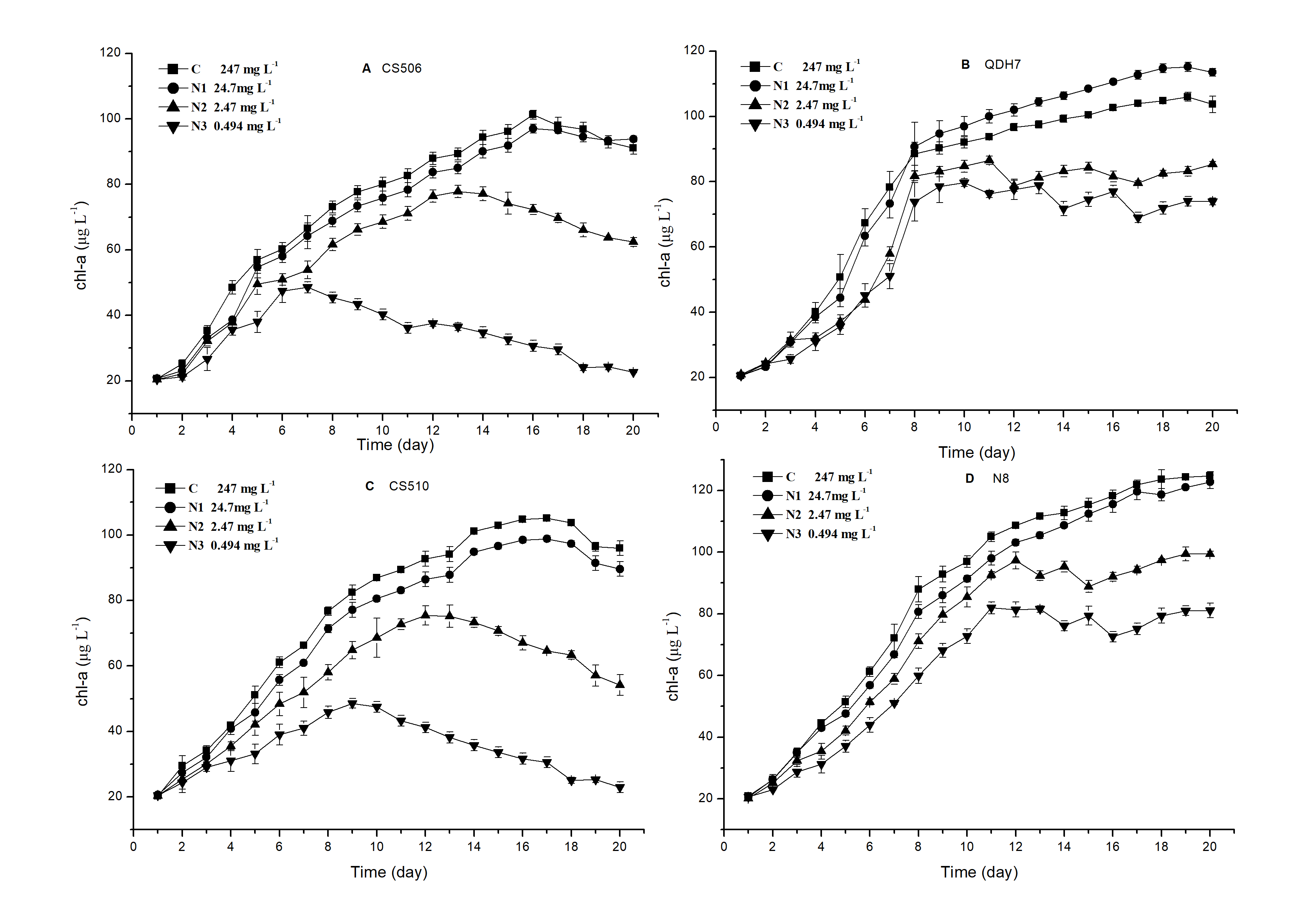


**FIGURE S2** Growth curves for two Australian (CS506 and CS510) and two Chinese (QDH7 and N8) strains of *R. raciborskii* under different nitrogen concentrations (BG11 control, N1, N2, and N3) in the monoculture experiments.


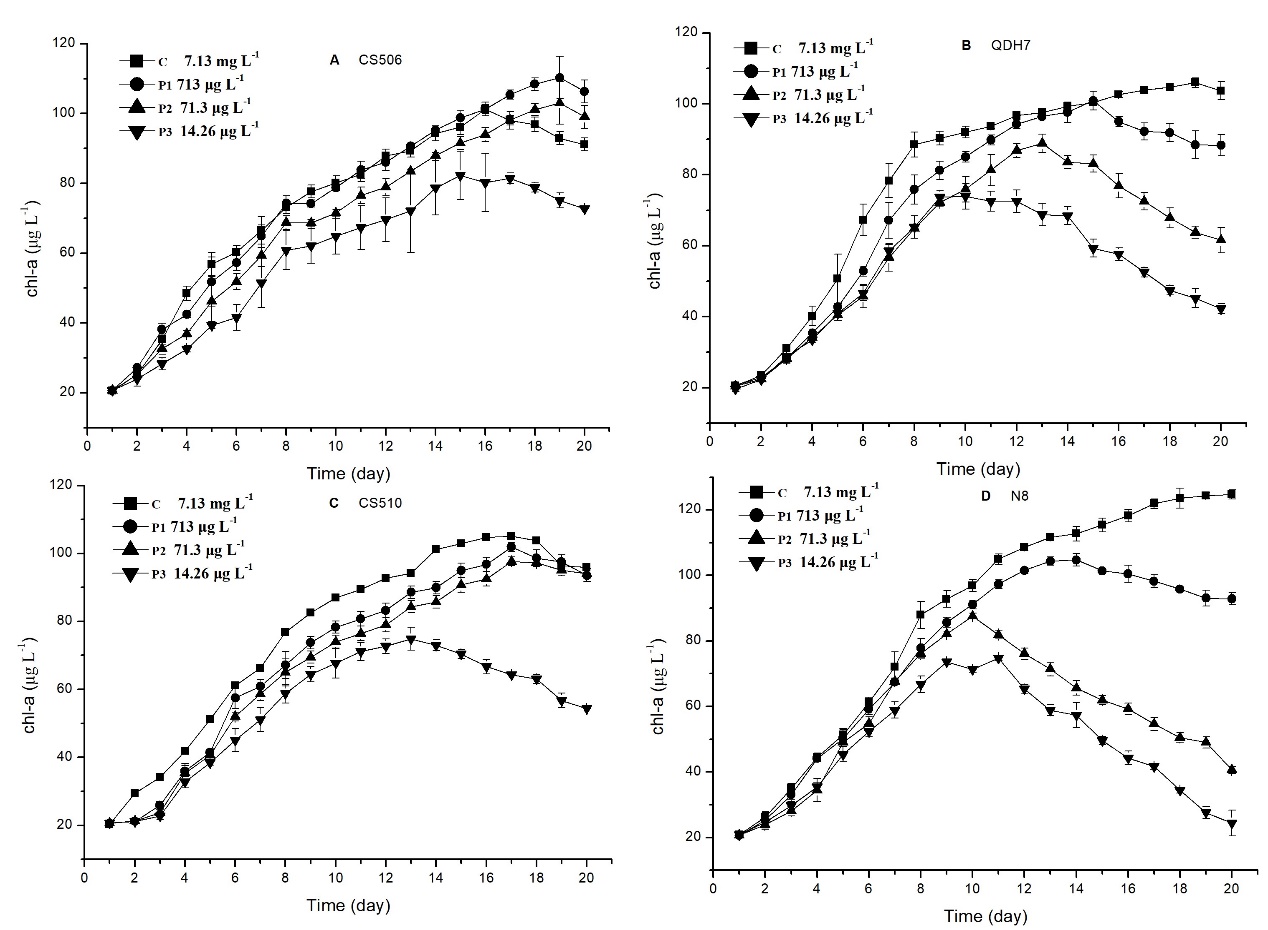


**FIGURE S3** Growth curves for two Australian (CS506 and CS510) and two Chinese (QDH7 and N8) strains of *R. raciborskii* under different phosphorus concentrations (BG11 control, P1, P2 and P3) in the monoculture experiments.
